# Supplementary material for: Modulation of Toll-like receptor 1 intracellular domain structure and activity by Zn2+ ions
Source: Commun Biol. 2021 Aug 24;4:1003. doi: 10.1038/s42003-021-02532-0 (PMC8385042; doi:10.1038/s42003-021-02532-0)
Supplement: Supplementary file 2 — Supplementary Information [file 42003_2021_2532_MOESM2_ESM.pdf]

Supplementary information

Modulation of toll-like receptor 1 intracellular domain structure and activity by Zn2+ ions.

Vladislav A. Lushpa[1,2], Marina V. Goncharuk[1], Cong Lin[3], Arthur O. Zalevsky[1], Irina A. Talyzina[1,4], Alexandra P. Luginina[2], Daniil D. Vakhrameev[2], Mikhail B. Shevtsov[2], Sergey A. Goncharuk[1,2], Alexander S. Arseniev[1], Valentin I. Borshchevskiy[2,5,6], Xiaohui Wang[3,7], and Konstantin S. Mineev[1,2*]

[*]corresponding author: mineev@nmr.ru

[1]Shemyakin-Ovchinnikov Institute of Bioorganic Chemistry RAS, Moscow 117997, Russia

[2]Moscow Institute of Physics and Technology, Dolgoprudny 141701, Russia

[3]Laboratory of Chemical Biology, Changchun Institute of Applied Chemistry, Chinese Academy of Sciences, Changchun, Jilin 130022, China

[4]Center of Life Sciences, Skolkovo Institute of Science and Technology, Moscow 121205, Russia

[5]Institute of Biological Information Processing (IBI-7: Structural Biochemistry), Forschungszentrum Jülich GmbH, 52425 Jülich, Germany

[6]JuStruct: Jülich Center for Structural Biology, Forschungszentrum Jülich GmbH, 52425 Jülich, Germany

[7]Department of Applied Chemistry and Engineering, University of Science and Technology of China, Hefei, 230026, China

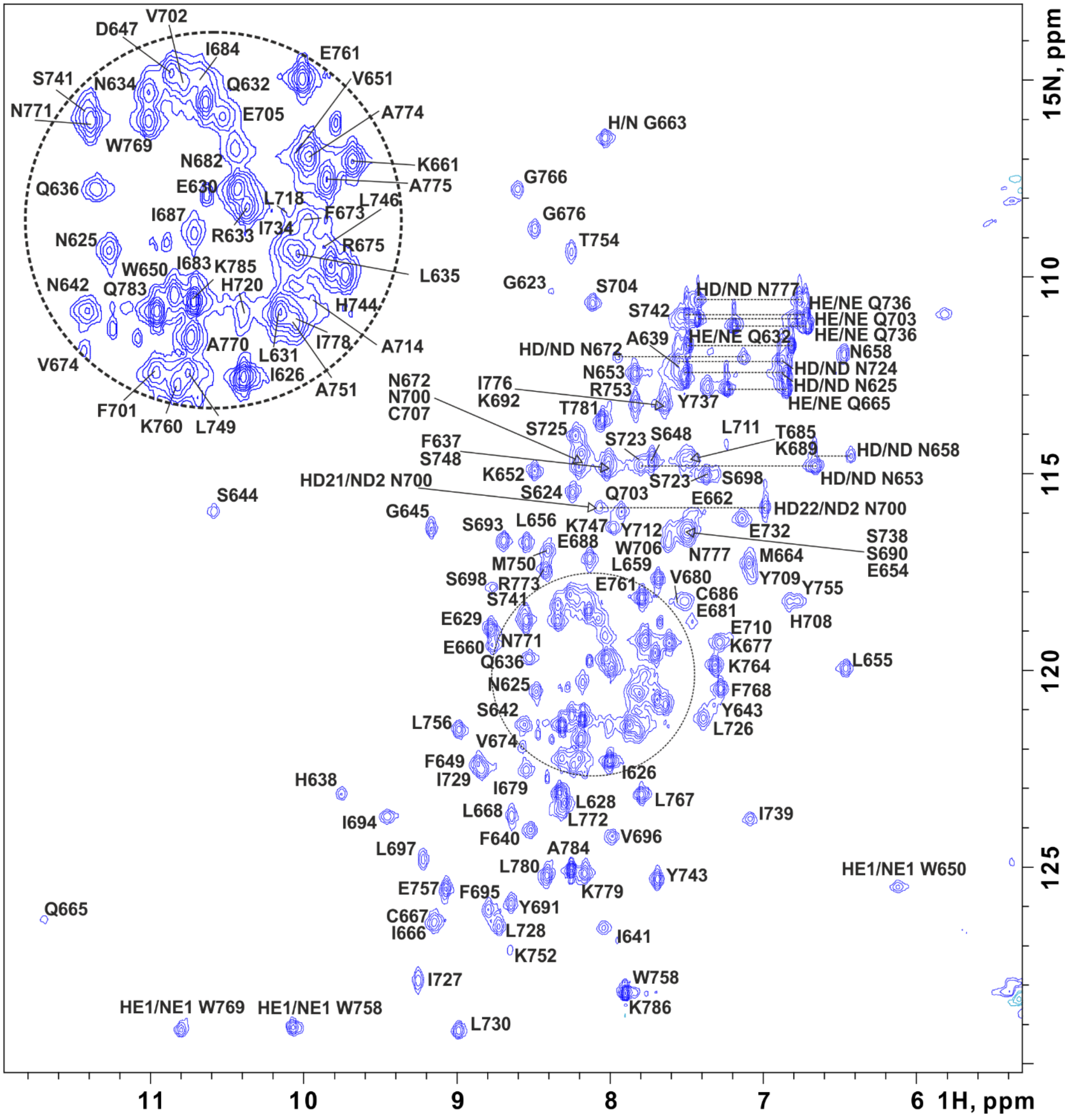

Supplementary Figure 1. $^{1}H,^{15}N$-HSQC NMR spectrum of TLR1-TIR recorded at 35 ºC, pH 6.3.

The assignment of amide groups is indicated.

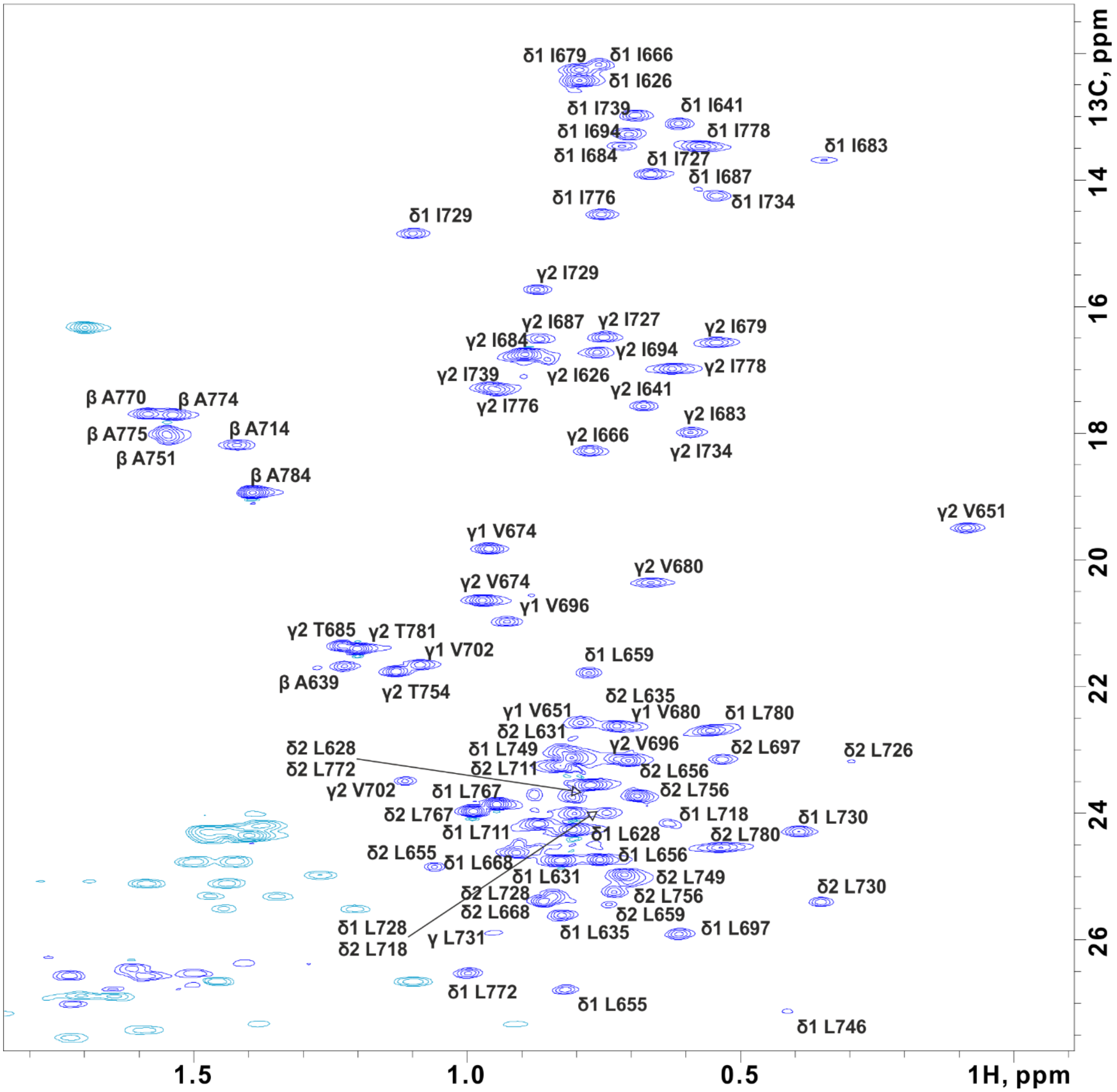

Supplementary Figure 2. A fragment of $^1H,^{13}C$-CT-HSQC NMR spectrum of TLR1-TIR recorded at 35 ^oC, pH 6.3. The assignment of methyl groups is indicated.

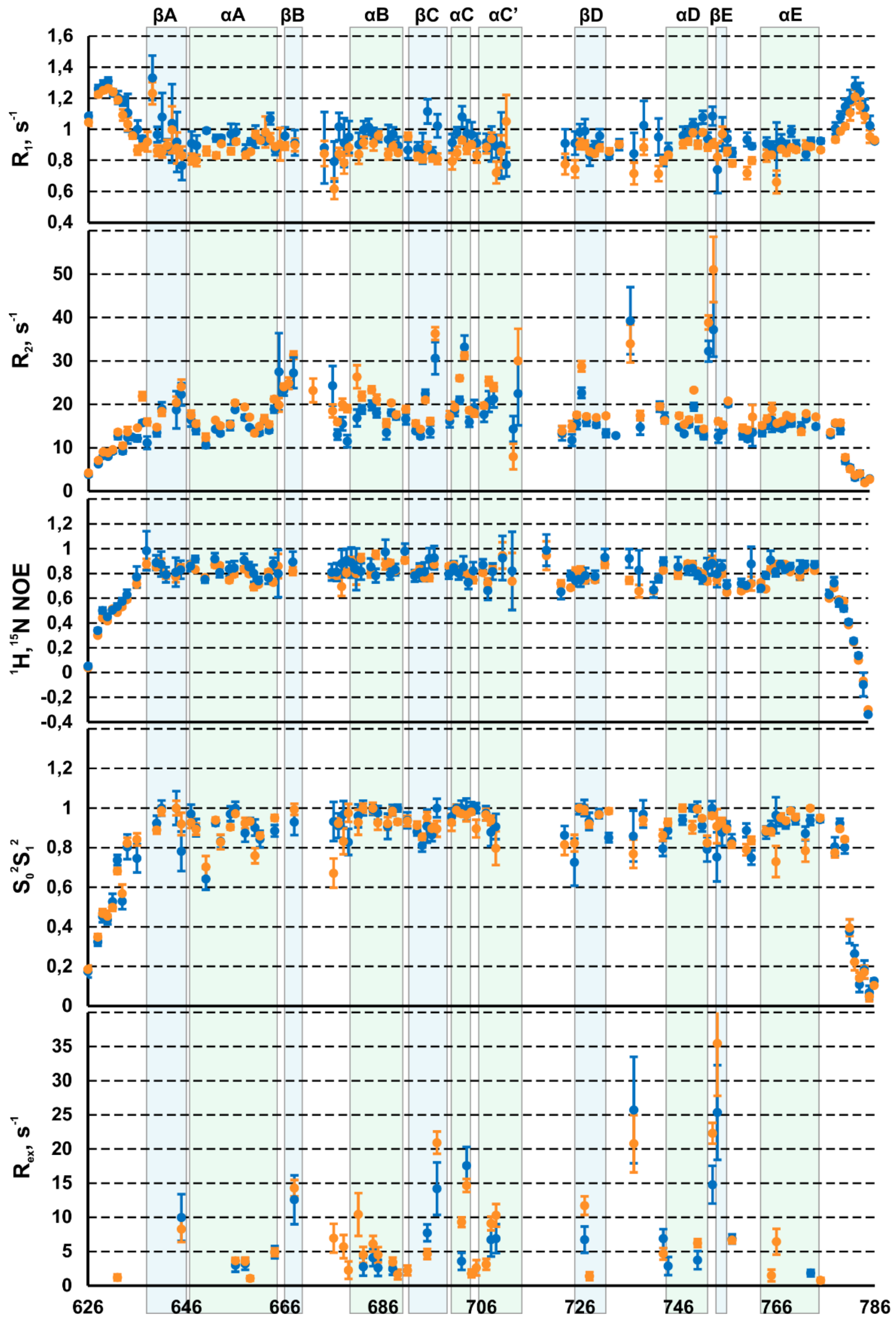

Supplementary Figure 3. NMR relaxation parameters of ^{15}N nuclei (rates of longitudinal (R1) and transverse (R2) relaxation, heteronuclear equilibrium NOE (^{1}H,^{15}N NOE)) and internal mobility parameters - generalized order parameter $S_0^2S_1^2$ and exchange contribution to the transverse relaxation R_{ex}, measured at two concentrations (blue corresponds to 3.3 mg/ml and orange - to 10.3 mg/ml).

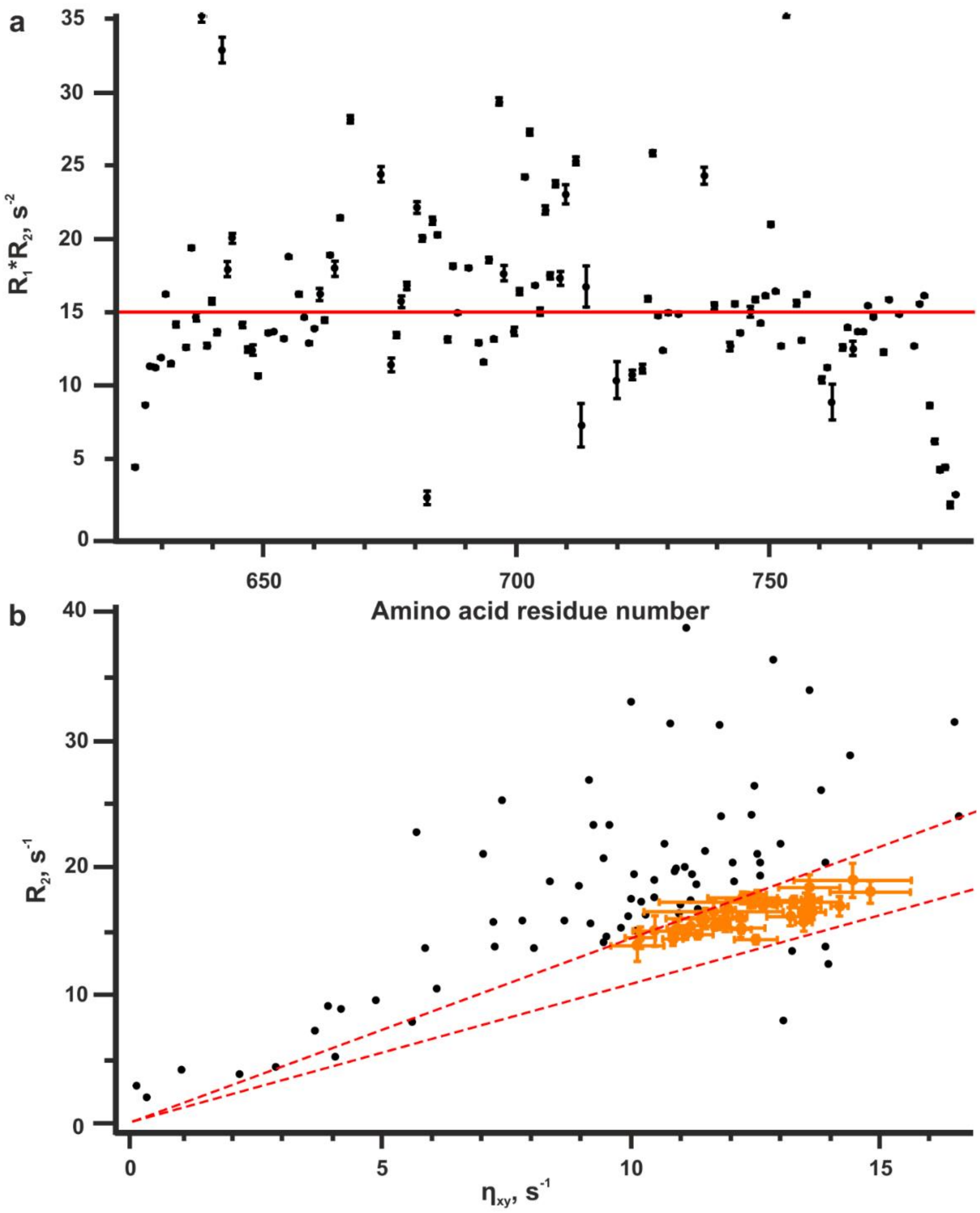

Supplementary Figure 4. Analysis of TLR1-TIR ^{15}N NMR relaxation. **a)** The R_1R_2 product. Orange line represents 15 s^{-2}, theoretical maximal for this parameter at 600 MHz in the absence of slow motions. Residues with R_1R_2 above the line are experiencing the motions in the us-ms timescale. **b)** Correlation plot of R_2 and η_{xy}. Region between two orange lines represents the theoretically possible values in the absence of slow motions. These residues were selected to calculate the rotational correlation time value. Points outside the specified region correspond to the residues, experiencing the motions in the us-ms timescale.

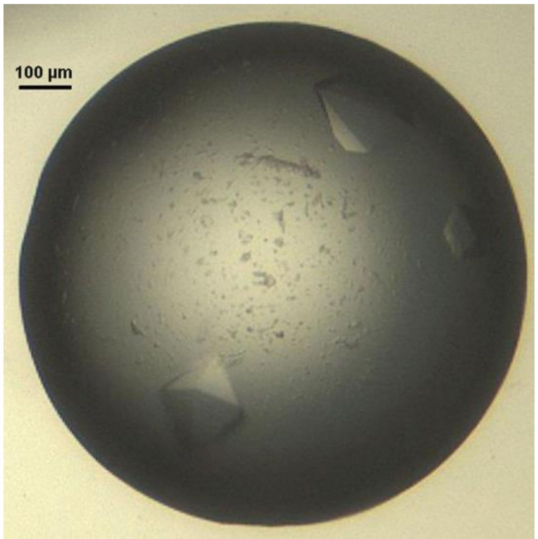

Supplementary Figure 5. Crystals of TLR1-TIR of $P6_222$ space group.

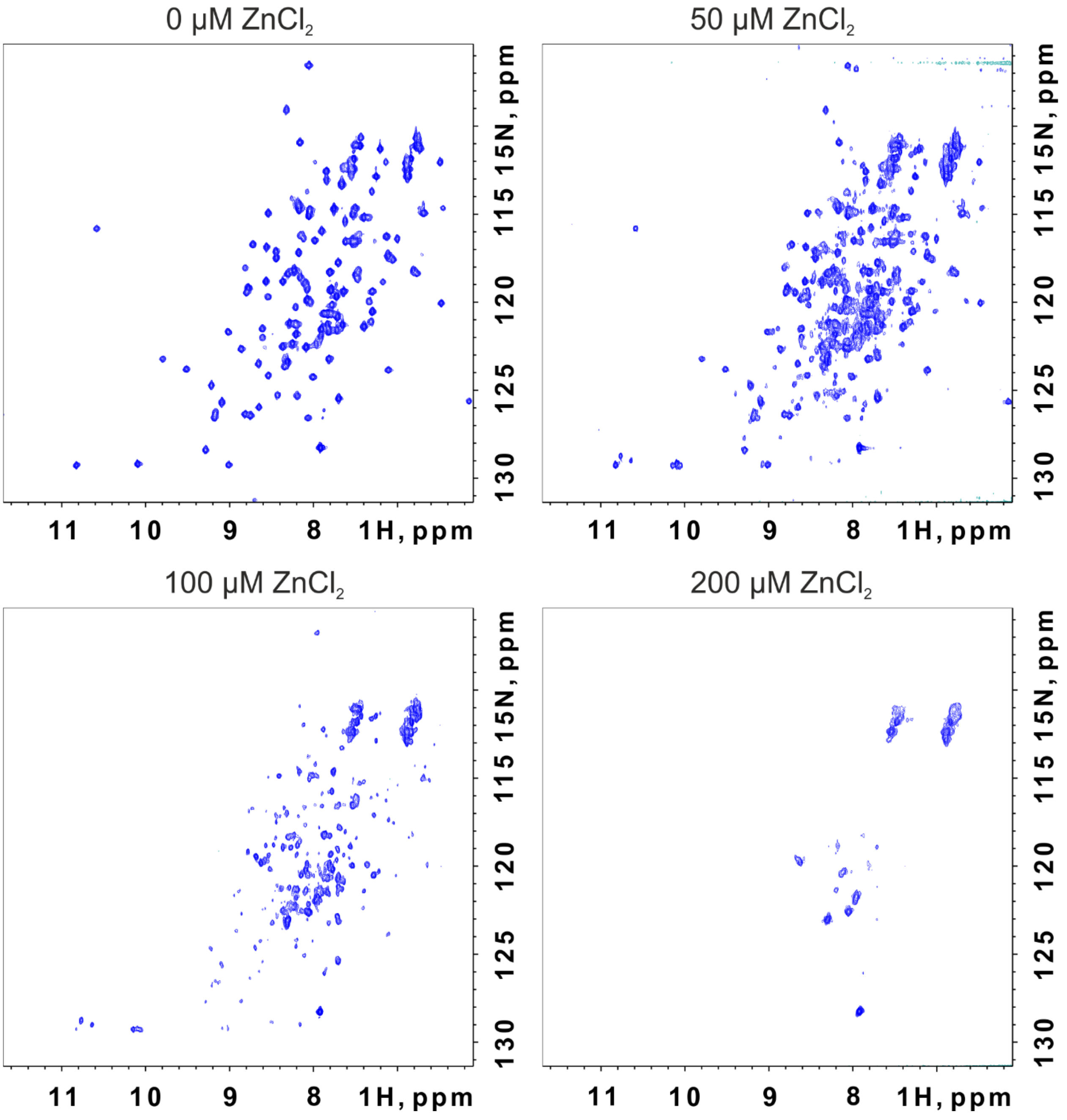

Supplementary Figure 6. ^{1}H,^{15}N-HSQC NMR spectra of 100 µM TLR1-TIR recorded at 35 ^{o}C, pH 7.4 in the presence of 0/50/100/200 µM of Zn^{2+} ions.

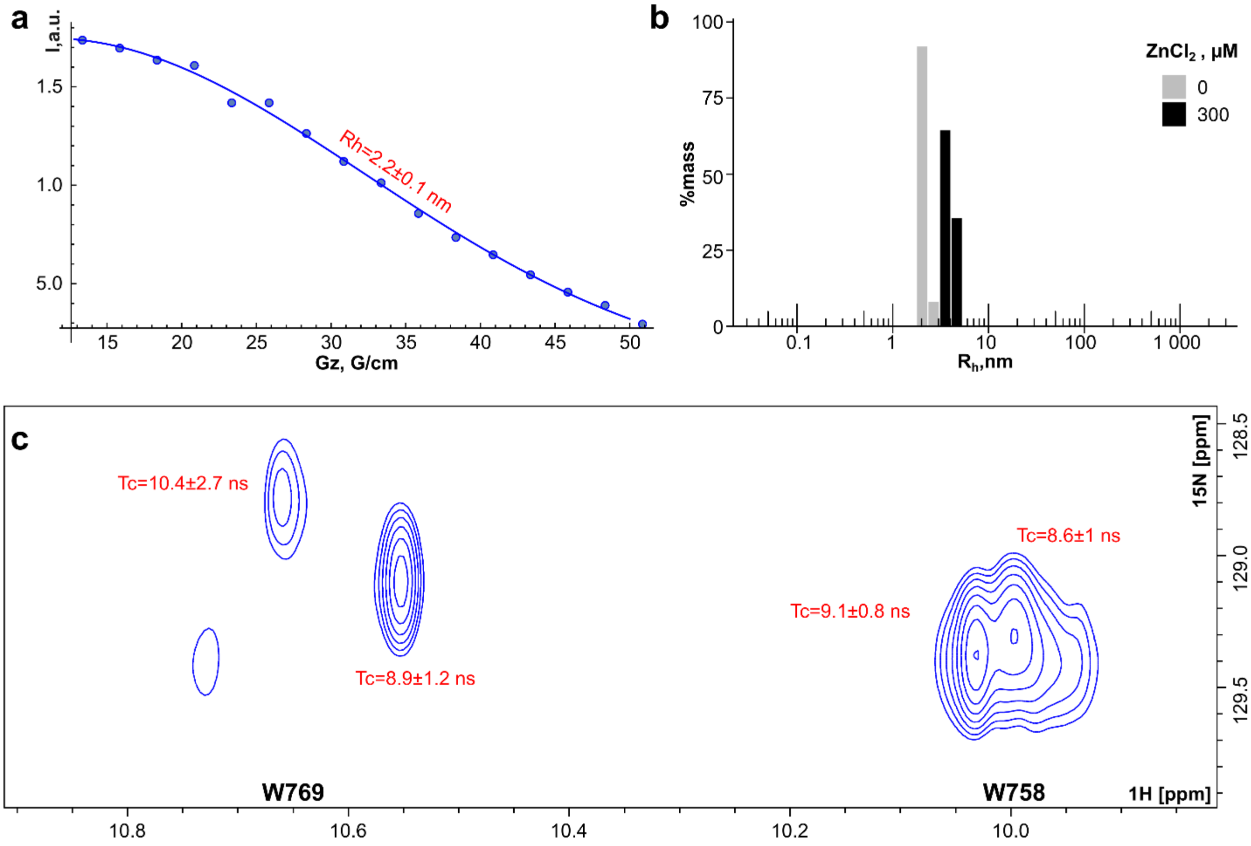

Supplementary Figure 7. Hydrodynamic properties of the TLR1-TIR in the presence and in the absence of Zn^{2+} ions. **a)** Intensity of NMR signal I as a function of gradient power Gz in the PGSTE-watergate experiment recorded for the 100 μM sample of TLR1-TIR in the absence of $ZnCl_2$ (pH 7.4, 30 ^{o}C). The decay is approximated by a theoretical dependence, shown by the solid blue line. Hydrodynamic radius, corresponding to the measured diffusion coefficient ($124{\cdot}10^{-12}$ m^2s^{-1}) is indicated. **b)** Mass-weighted distributions of hydrodynamic radii measured by DLS for the 100 μM sample of TLR1-TIR in the absence of Zn^{2+} ions (gray bars) and in the presence of 300 μM $ZnCl_2$ (black bars). **c)** Fragment of the reference TROSY-HSQC spectrum recorded for the 200 μM TLR1-TIR/Zn 1:1 sample that was used to measure the cross-correlated relaxation rates and estimate the rotational diffusion. Spectrum was recorded at pH 7.4 and 30 ^{o}C overnight. Correlation times of rotational diffusion obtained for each cross-peak of the Zn-bound TLR1-TIR are indicated.

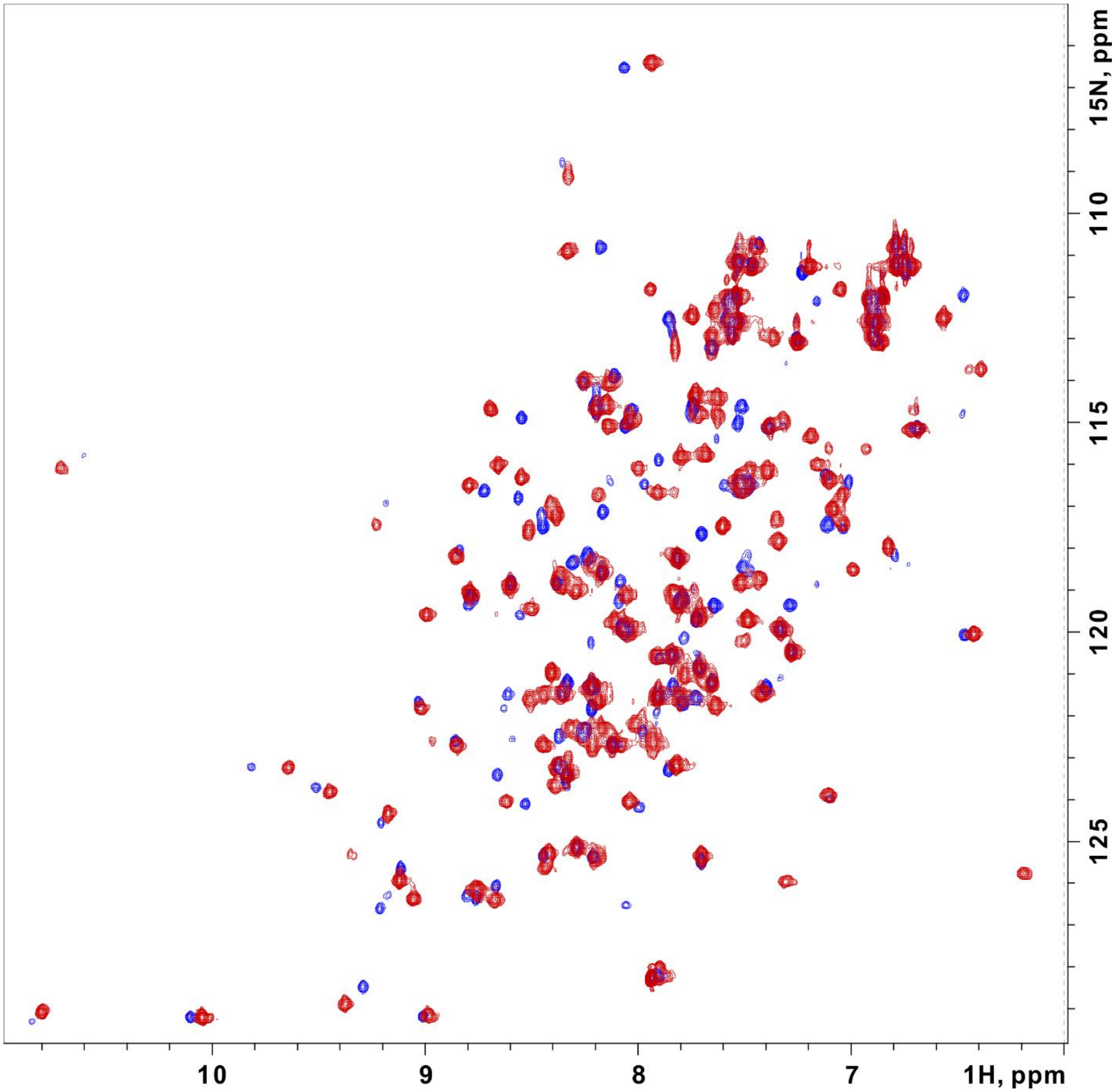

Supplementary Figure 8. Overlay of ^{1}H,^{15}N-HSQC NMR spectra of 100 μM TLR1-TIR recorded at 30 ^{o}C, pH 7.4 in the presence of 100 μM Co^{2+} ions (red) and without them (blue).

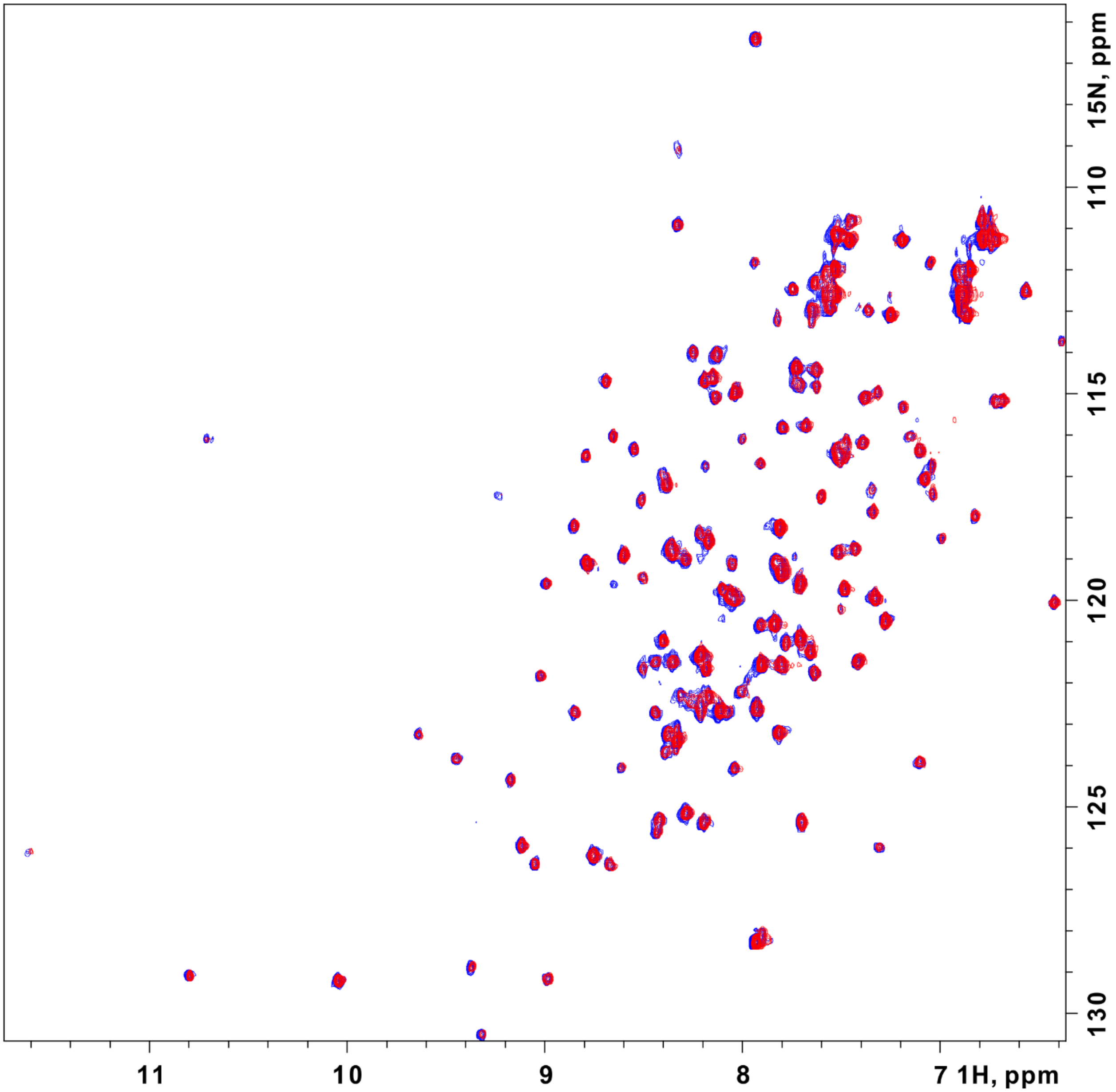

Supplementary Figure 9. Overlay of $^{1}H,^{15}N$-HSQC NMR spectra of 100 μM TLR1-TIR recorded at 30 °C, pH 7.4 in the presence of 100 μM Co^{2+} ions (blue) or 100 μM of Cu^{2+} ions (red).

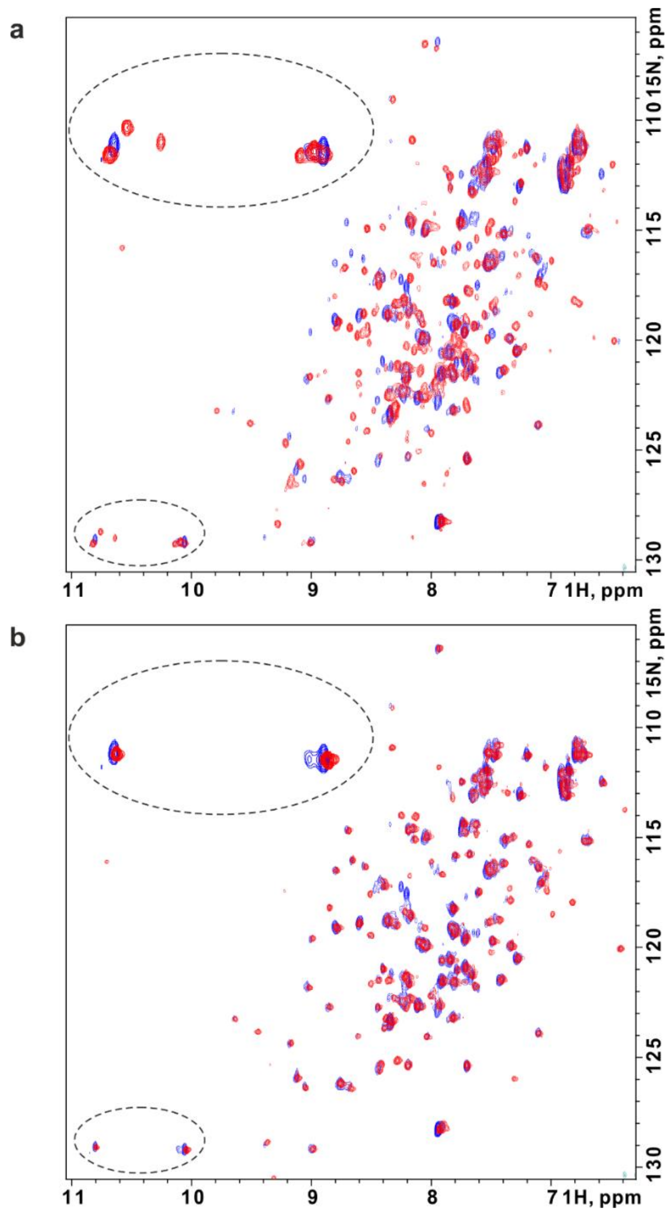

Supplementary Figure 10. The influence of Zn^{2+} and Co^{2+} ions on TLR1-TIR disulfide bond formation. **a)** Overlay of ^{1}H,^{15}N-HSQC NMR spectra of 100 μM TLR1-TIR recorded at 30 °C, pH 7.4 after the treatment with GSH/GSSG overnight (shown in blue, cysteines oxidized) and after the addition of 50 μM $ZnCl_2$ (shown in red). Spectra are different, the zinc binding does not cause the disulfide formation. **b)** Overlay of ^{1}H,^{15}N-HSQC NMR spectra of 100 μM TLR1-TIR recorded at 30 °C, pH 7.4 after the treatment with GSH/GSSG overnight (shown in blue, cysteines oxidized) and after the addition of 50 μM $CoCl_2$ (shown in red). Co^{2+} catalyzes the disulfide formation in TLR1-TIR.

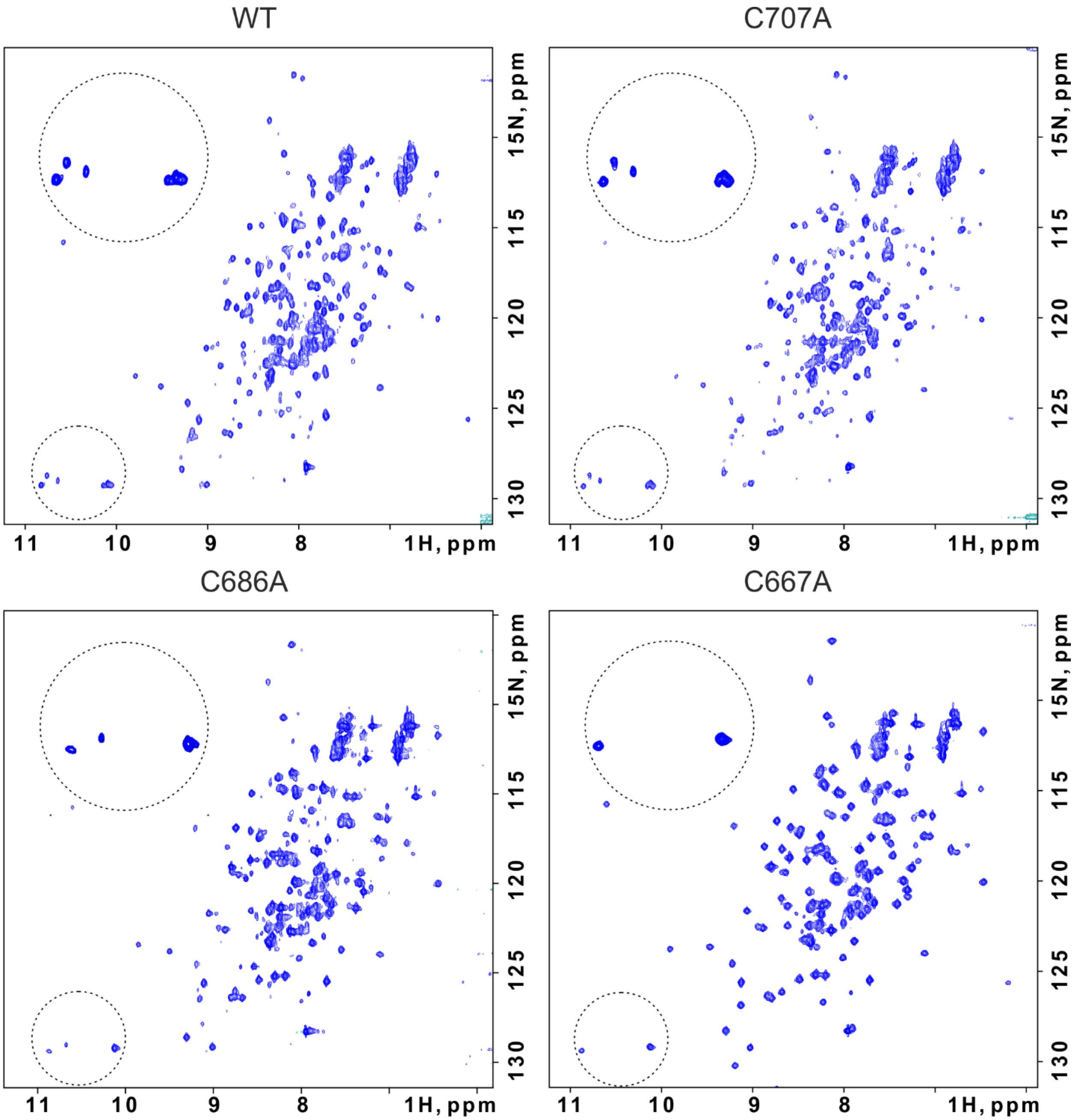

Supplementary Figure 11. $^{1}H,^{15}N$-HSQC spectra of TLR1-TIR wild-type and of three cysteine mutants, recorded for the 100 μM TLR1-TIR at 30 °C, pH 7.4 in the presence of 50 μM $ZnCl_2$. Signals from the Trp side chains, which were used for the analysis are highlighted by a dashed circle and additionally provided at a larger size at the left upper corner of each spectrum.

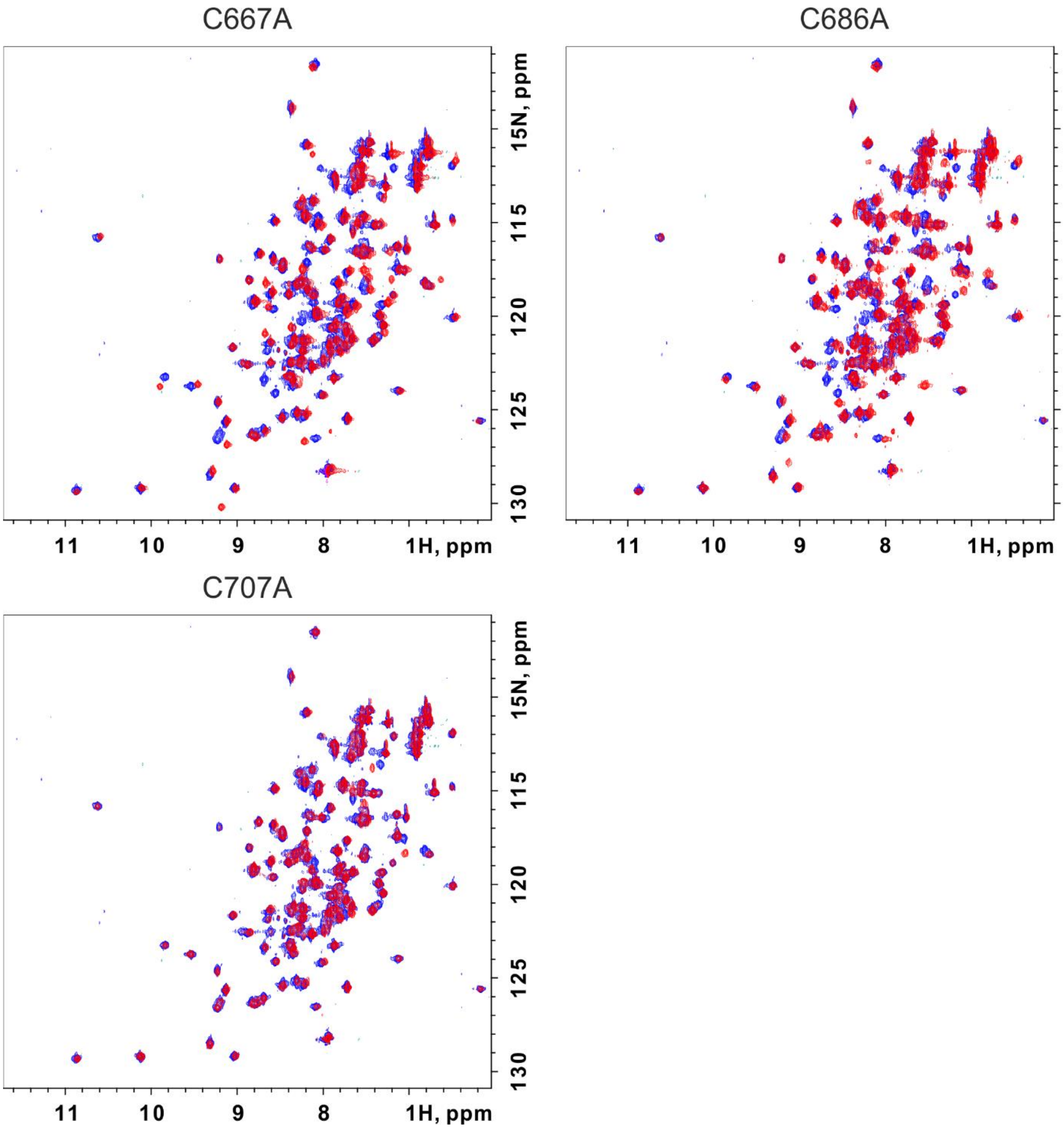

Supplementary Figure 12. Overlay of ^{1}H,^{15}N-HSQC spectra of TLR1-TIR wild-type (blue) and of three cysteine mutants (red), recorded for the 100 μM TLR1-TIR at 30 ^{o}C, pH 7.4 in the absence of $ZnCl_2$.

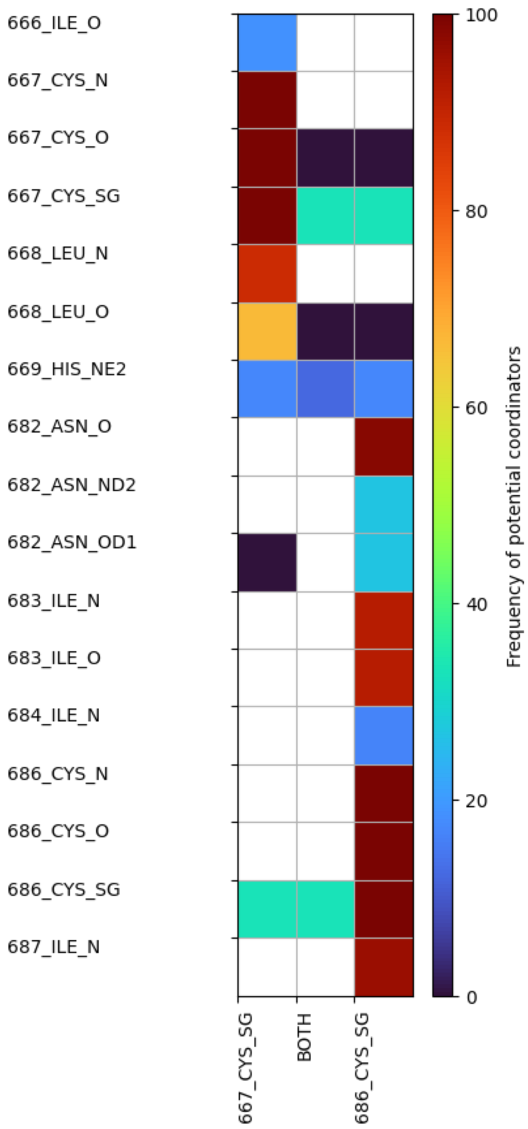

Supplementary Figure 13. Normalized frequencies of conformations with distances between possible Zn2+ coordinators less than 5.0Å. Total 2000 conformations (100 loop conformations for every NMR structure) were sampled with Rosetta Loopmodel protocol. Only atoms with frequencies >15 are shown.

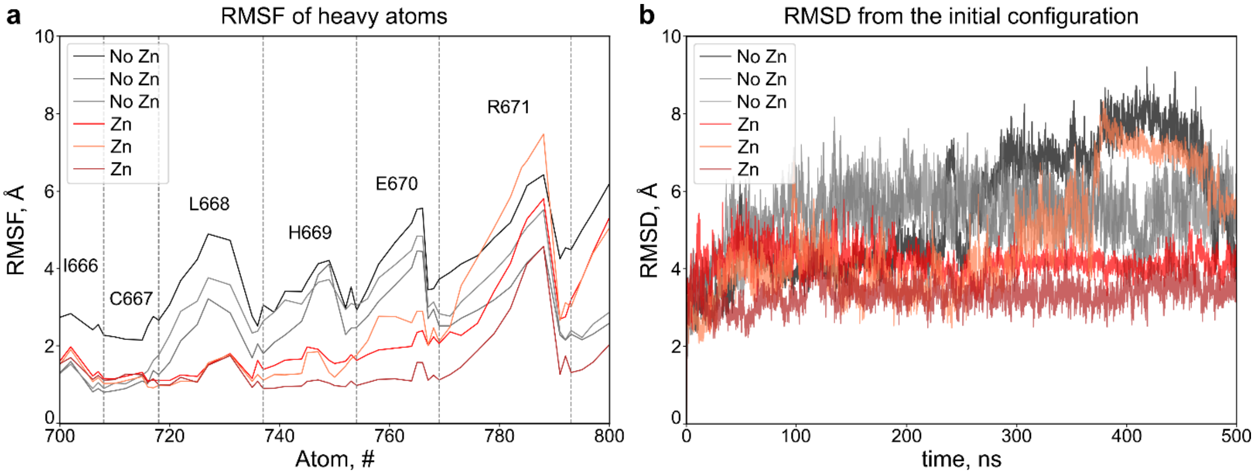

Supplementary Figure 14. The properties of **Zn1** state. **a)** Root Mean Square Fluctuations (RMSF) for systems with C667-H669-C686 coordination mode. The most significant differences (up to 3Å) between two types of systems are concentrated around the region I666 - E670. Only heavy atoms (except hydrogens) are considered. **b)** Root Mean Square Displacement (RMSD) of the BB-loop over the course of simulation. Only heavy atoms (except hydrogens) are considered.

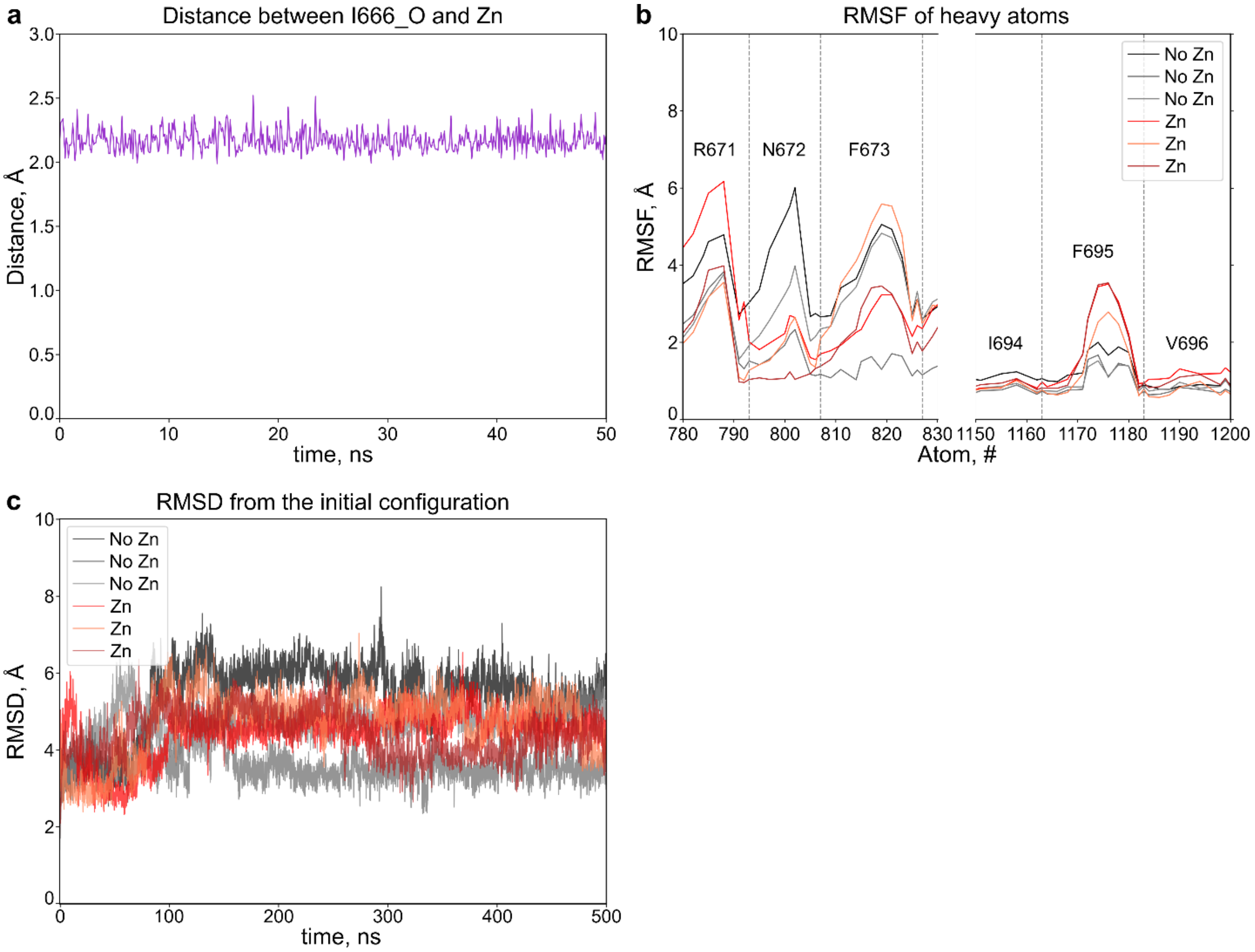

Supplementary Figure 15. The properties of **Zn2** state. **a)** Distance between I666_O and Zn in the unrestrained test simulation. The coordination is stable even without specialized parameters for the Zn coordination by the backbone oxygen. **b)** Root Mean Square Fluctuations (RMSF) for systems with the C6667-I666 backbone coordination mode in restrained simulations. The most significant differences (up to 3Å) between two types of systems are in the areas of R671_O which also joins the coordination sphere and F695 buried in the hydrophobic core. Only heavy atoms (except hydrogens) are considered. **c)** Root Mean Square Displacement (RMSD) of the BB-loop over the course of simulation. Only heavy atoms (except hydrogens) are considered.

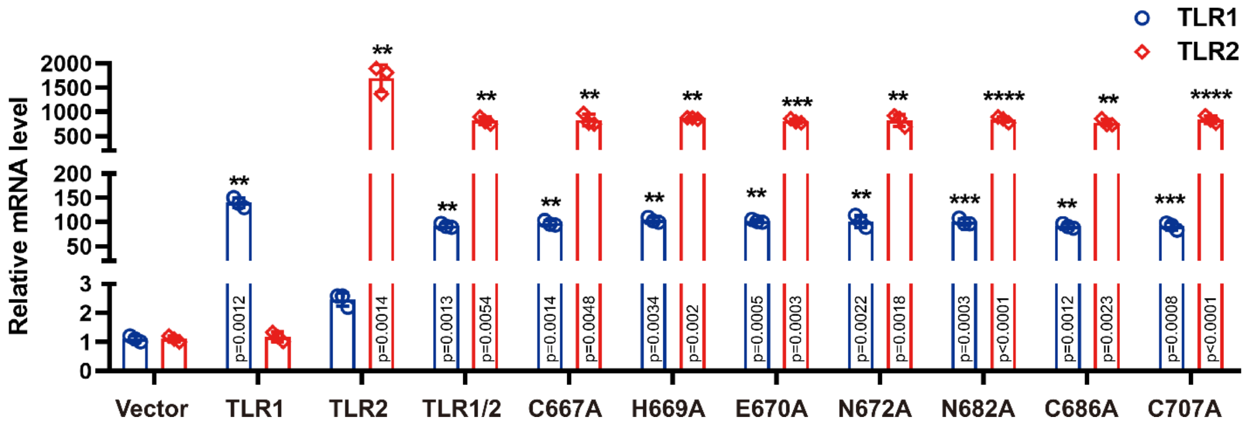

Supplementary Figure 16. TLR1 and TLR2 mRNA levels were assessed by qRT-PCR in HEK Blue 293 cells expressing human TLR1 or TLR2 alone and co-expressing the wild-type or mutant human TLR1 and TLR2. Statistical significance is indicated as follows: *, **, *** and **** - p<0.05, p<0.01, p<0.001 and p<0.0001 with respect to the negative control. Error bars represent the standard error of the mean.

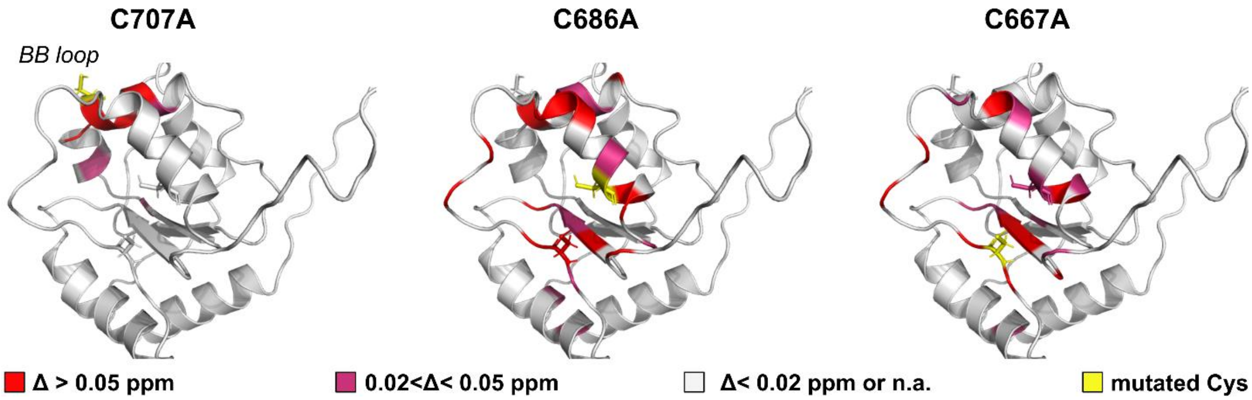

Supplementary Figure 17. NMR chemical shift changes observed for the amide groups of TLR1-TIR (**Δ**), due to three Cys to Ala mutations are mapped on the spatial structure of the domain. Red regions correspond to the substantial changes, exceeding 0.05 ppm in proton dimension. Magenta regions correspond to the residues with the moderate changes and gray regions denote the residues with no changes observed (or with no data available). Sidechain of the mutated Cys residue is shown in yellow.
