# Supplementary material for: Modulation of Toll-like receptor 1 intracellular domain structure and activity by Zn2+ ions
Source: Commun Biol. 2021 Aug 24;4:1003. doi: 10.1038/s42003-021-02532-0 (PMC8385042; doi:10.1038/s42003-021-02532-0)
Supplement: Supplementary file 3 — Description of Additional Supplementary Files [file 42003_2021_2532_MOESM3_ESM.pdf]

Description of Additional Supplementary Files

File name: Supplementary Data 1

Description: The raw data from functional assay.
