# Supplementary material for: Modulation of Toll-like receptor 1 intracellular domain structure and activity by Zn2+ ions
Source: Commun Biol. 2021 Aug 24;4:1003. doi: 10.1038/s42003-021-02532-0 (PMC8385042; doi:10.1038/s42003-021-02532-0)
Supplement: Supplementary file 5 — Reporting Summary [file 42003_2021_2532_MOESM5_ESM.pdf]

nature portfolio

Corresponding author(s): Konstantin S Mineev

Last updated by author(s): Jul 21, 2021

Reporting Summary

Nature Portfolio wishes to improve the reproducibility of the work that we publish. This form provides structure for consistency and transparency in reporting. For further information on Nature Portfolio policies, see our Editorial Policies and the Editorial Policy Checklist.

Statistics

For all statistical analyses, confirm that the following items are present in the figure legend, table legend, main text, or Methods section.

n/a	Confirmed	
☐	☒	The exact sample size (*n*) for each experimental group/condition, given as a discrete number and unit of measurement
☐	☒	A statement on whether measurements were taken from distinct samples or whether the same sample was measured repeatedly
☐	☒	The statistical test(s) used AND whether they are one- or two-sided<br>*Only common tests should be described solely by name; describe more complex techniques in the Methods section.*
☒	☐	A description of all covariates tested
☒	☐	A description of any assumptions or corrections, such as tests of normality and adjustment for multiple comparisons
☒	☐	A full description of the statistical parameters including central tendency (e.g. means) or other basic estimates (e.g. regression coefficient) AND variation (e.g. standard deviation) or associated estimates of uncertainty (e.g. confidence intervals)
☐	☒	For null hypothesis testing, the test statistic (e.g. *F*, *t*, *r*) with confidence intervals, effect sizes, degrees of freedom and *P* value noted<br>*Give P values as exact values whenever suitable.*
☒	☐	For Bayesian analysis, information on the choice of priors and Markov chain Monte Carlo settings
☒	☐	For hierarchical and complex designs, identification of the appropriate level for tests and full reporting of outcomes
☒	☐	Estimates of effect sizes (e.g. Cohen's *d*, Pearson's *r*), indicating how they were calculated

Our web collection on statistics for biologists contains articles on many of the points above.

Software and code

Policy information about availability of computer code

Data collection	Topspin 3.0 (Bruker Biospin) software was used to collect the NMR data. DYNAMICS 6.7.6 was used to collect the DLS data. Analytik Jena qPCRsoft 4.0 was used to collect the qPCR data. Gen5 microplate reader software was used to collect the SEAP data.
Data analysis	CARA 1.9.3, CYANA 3.9.8, mddNMR 2.7 and TENSOR 2.0 were used to analyze the NMR data. DYNAMICS 6.7.6 was used to analyze the DLS data. XDS, autoPROC, Phaser and PHENIX, PHENIX.AutoBuild, PHENIX.Refine and Coot software packeges were used to analyze the X-ray data. Analytik Jena qPCRsoft 4.0 and GraphPad Prim 5 were used to analyze the qPCR data. GraphPad Prim 5 were used to analyze the SEAP data.

For manuscripts utilizing custom algorithms or software that are central to the research but not yet described in published literature, software must be made available to editors and reviewers. We strongly encourage code deposition in a community repository (e.g. GitHub). See the Nature Portfolio guidelines for submitting code & software for further information.

Data

Policy information about availability of data

All manuscripts must include a data availability statement. This statement should provide the following information, where applicable:

- Accession codes, unique identifiers, or web links for publicly available datasets
- A description of any restrictions on data availability
- For clinical datasets or third party data, please ensure that the statement adheres to our policy

NMR structure of the TLR1-TIR, peak lists and chemical shifts were deposited to the PDB under the access code 7NT7 and to BMRB under the access number 34610. The structures for the TLR1-TIR crystallised with and without Zn were deposited in Protein Data Bank with accession numbers 7NUX and 7NUW, respectively.

Field-specific reporting

Please select the one below that is the best fit for your research. If you are not sure, read the appropriate sections before making your selection.

☒ Life sciences ☐ Behavioural & social sciences ☐ Ecological, evolutionary & environmental sciences

For a reference copy of the document with all sections, see nature.com/documents/nr-reporting-summary-flat.pdf

Life sciences study design

All studies must disclose on these points even when the disclosure is negative.

Sample size	We chose the minimal sample size, which is sufficient to perform a statistical test
Data exclusions	No data were excluded from the analysis in SEAP experiments. Data points with extremely large particles (mkM) and detector overflow were excluded from the DLS analysis.
Replication	NMR experiments on Zinc binding were repeated several times under various conditions. To ensure reproducibility of the findings, for all cell assays, at least three independent biological replicates were performed (n=3).
Randomization	Cells were distributed randomly between the experiments
Blinding	Blinding is not applicable to the current study

Reporting for specific materials, systems and methods

We require information from authors about some types of materials, experimental systems and methods used in many studies. Here, indicate whether each material, system or method listed is relevant to your study. If you are not sure if a list item applies to your research, read the appropriate section before selecting a response.

Materials & experimental systems

n/a	Involved in the study	
☒	☐	Antibodies
☐	☒	Eukaryotic cell lines
☒	☐	Palaeontology and archaeology
☒	☐	Animals and other organisms
☒	☐	Human research participants
☒	☐	Clinical data
☒	☐	Dual use research of concern

Methods

n/a	Involved in the study	
☒	☐	ChIP-seq
☒	☐	Flow cytometry
☒	☐	MRI-based neuroimaging

Eukaryotic cell lines

Policy information about cell lines

Cell line source(s)	HEK Blue 293 cells were purchased from InvivoGen.
Authentication	The cell lines are authenticated by manufacturer (InvivoGen)
Mycoplasma contamination	Cells were routinely checked the mycoplasma contamination by Mycoplasma PCR detection kit before the experiments. The mycoplasma-positive cell ine will be discard.
Commonly misidentified lines (See ICLAC register)	No commonly misidentified lines were used in this study.
